# Phytotherapy for Attention Deficit Hyperactivity Disorder (ADHD): A Systematic Review and Meta-analysis

**DOI:** 10.3389/fphar.2022.827411

**Published:** 2022-05-03

**Authors:** Tusheema Dutta, Uttpal Anand, Shreya Sikdar Mitra, Mimosa Ghorai, Niraj Kumar Jha, Nusratbanu K. Shaikh, Mahipal S Shekhawat, Devendra Kumar Pandey, Jarosław Proćków, Abhijit Dey

**Affiliations:** ^1^ Department of Life Sciences, Presidency University, Kolkata, India; ^2^ CytoGene Research & Development LLP, Lucknow, Uttar Pradesh, India; ^3^ Department of Biotechnology, School of Engineering & Technology, Sharda University, Greater Noida, India; ^4^ Department of Pharmaceutical Chemistry, Smt N. M. Padalia Pharmacy College, Ahmedabad, India; ^5^ Department of Plant Biology and Biotechnology, Kanchi Mamunivar Government Institute for Postgraduate Studies and Research, Lawspet, India; ^6^ Department of Biotechnology, School of Biosciences, Lovely Professional University, Phagwara, India; ^7^ Department of Plant Biology, Institute of Environmental Biology, Wrocław University of Environmental and Life Sciences, Wrocław, Poland

**Keywords:** herbal treatments, attention deficit hyperactivity disorder (ADHD), complementary alternative medicine (CAM), phytotherapy, Melissa officinalis L., Valeriana officinalis L

## Abstract

Attention deficit hyperactivity disorder (ADHD) is commonly a neurodevelopmental behavioural disorder in children and adolescents. Mainly characterized by symptoms like lack of attention, hyperactivity, and impulsiveness, it can impact the overall mental development of the one affected. Several factors, both genetic and non-genetic, can be responsible for this disorder. Although several traditional treatment methods involve medication and other counselling techniques, they also come with different side effects. Hence, the choice is now shifting to alternative treatment techniques. Herbal treatments are considered one of the most popular complementary and alternative medicine (CAM) administered. However, issues related to the safety and efficacy of herbal remedies for the treatment of ADHD need to be investigated further. This study aims to find out the recent advancement in evidence-based use of herbal remedies for ADHD by a comprehensive and systematic review that depicts the results of the published works on herbal therapy for the disorder. The electronic databases and the references retrieved from the included studies present related randomized controlled trials (RCTs) and open-label studies. Seven RCTs involving children and adolescents diagnosed with ADHD met the inclusion criteria. There is a fair indication of the efficacy and safety of Melissa officinalis L., Bacopa monnieri (L.) Wettst., Matricaria chamomilla L., and Valeriana officinalis L. from the studies evaluated in this systematic review for the treatment of various symptoms of ADHD. Limited evidence was found for Ginkgo biloba L. and pine bark extract. However, various other preparations from other plants did not show significant efficacy. There is inadequate proof to strongly support and recommend the administration of herbal medicines for ADHD, but more research is needed in the relevant field to popularize the alternative treatment approach.

## 1 Introduction

ADHD is one of the most common neurodevelopmental behavioral disorders characterized by inattentiveness, hyperactivity, and impulsiveness (Datta et al., 2021). It is found amongst 1 out of 20 children (Pellow et al., 2011). Around 50% of children and adolescents with this disorder may still suffer from significant symptoms during their adulthood (Faraone and Biederman 2005). It often involves different symptoms such as lack of attention, inability to stay focused, impulsive actions, lack of self-motivation, and self-control. Different factors are found to be associated, such as nutritional, biochemical, and genetic. Nutritional deficiencies of various types, exposure to different environmental toxic products, overexposure to electronic media, and culture are various types of risk factors. Different types of conventional treatments available involve medications, behavior therapy, individual and family counseling. Conventional treatments, incredibly different medications, often involve side effects such as insomnia, decreased appetite, fatigue, hypertension, liver toxicity, nausea, and seizures. Hence, the preference is shifting to different types of conventional and alternative medicines (CAM) mainly for the price affordability and lesser side effects (Anand et al., 2019; Banerjee et al., 2021; Dutta et al., 2021; Anand et al., 2022).

### 1.1 Complementary and Alternative Medicine

CAM has been increasingly popularized, especially for the treatment of ADHD in children. Holistic and individual treatment offered by CAM attempts to diagnose and heal the underlying etiologies. Different popularly used CAM treatment methods involve dietary modifications, nutritional supplements, and different phytotherapeutic techniques. Hence, they often prefer options over traditional drug therapies due to their lower side effects and low price (Anand et al., 2020; Mohammed et al., 2021). Herbal medicine is one of the famous CAM. Its holistic approach to treating patients based on pathogenesis has popularized CAM even in treating ADHD (Pellow et al., 2011).

Diet, elimination diets, nutritional supplements (for example, supplementation with essential fatty acids), homeopathy, yoga, exercise, massage, and botanicals have been tested against ADHD (Pellow et al., 2011). Both single and multi-ingredient formulas have been reported effective in several studies, and combination therapies were found to be beneficial against cognitive and behavioral problems (Rucklidge et al., 2009; Banerjee et al., 2021; Datta et al., 2021; Halder et al., 2021; Tandon et al., 2021). However, the safety and efficacy of CAM treatments, especially for children, need to be further investigated.

## 2 Objective

This systematic review aims to evaluate the safe and effective use of various herbal remedies that are potential candidates as CAMs for the treatment of ADHD.

## 3 Methods

This review was conceptualized, organized, and executed according to the PRISMA (Preferred Reporting Items for Systematic Reviews and Meta-Analyses) recommendations and the Cochrane Collaboration guidelines (Higgins and Wells 2011).

### 3.1 Selection Criteria

**FIGURE 1:**
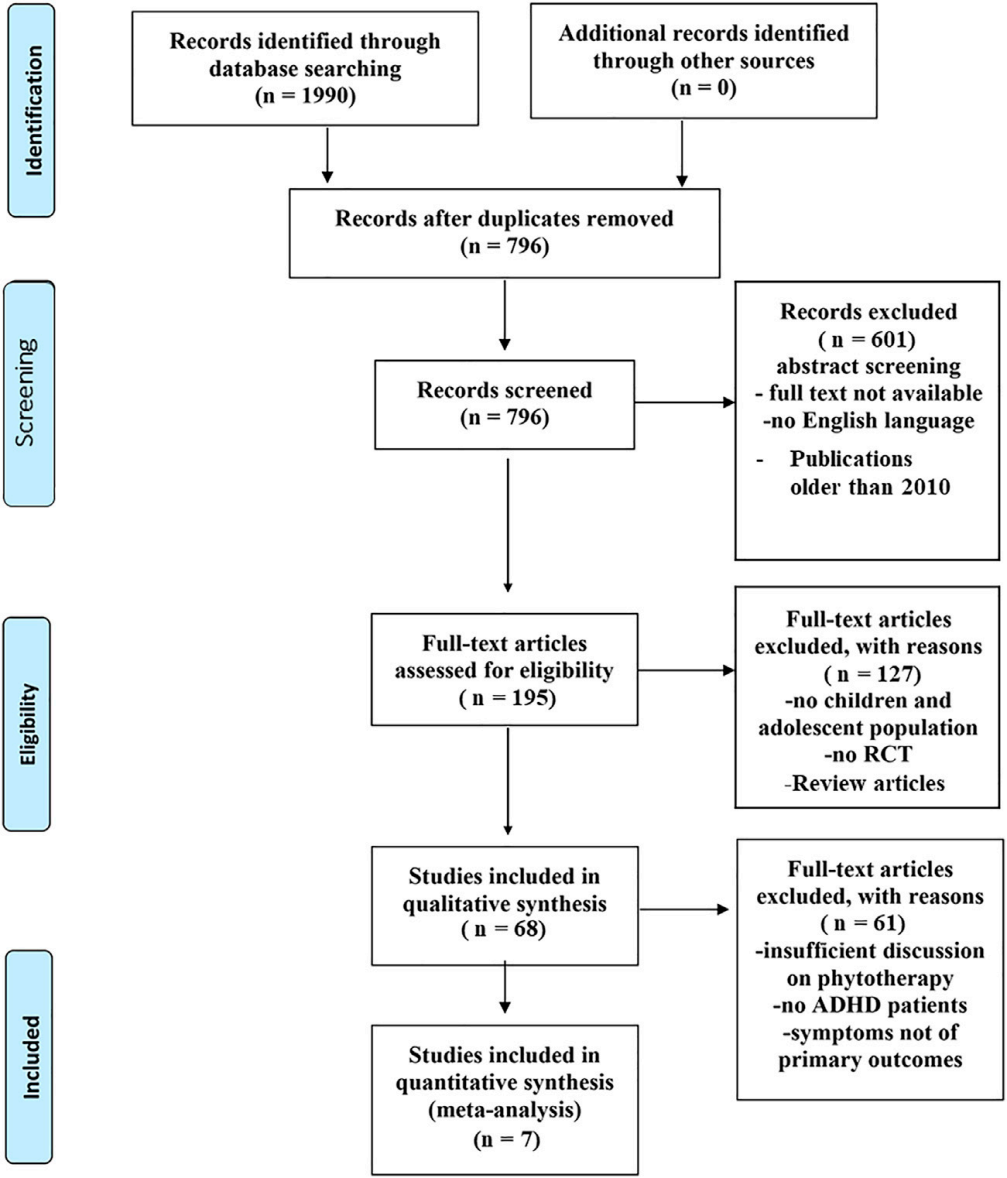
PRISMA Flowchart showing the literature search for the systemic review.

This review considered the following parameters as selection criteria for papers which have been shown in the form of a PRISMA flowchart (Figure 1):

(1) Firstly, all published randomized controlled trials (RCT) and open-label studies to evaluate the safe or effective use of herbal remedies for the treatment of ADHD were taken.
(2) Then only the papers which had children and adolescents generally between 0 and 18 years of age who showed symptoms of ADHD, as suggested by established diagnostic criteria (e.g., DSM-IV or Diagnostic and Statistical Manual of Mental Disorders) and different standardized instruments for measurement, as participants were considered. There were no restrictions on participant sex, trial, or their comorbidity.
(3) Only full-text publications in the English language have been included.
(4) Trials involving the administration of oral mono-preparations, bi-preparations, and compound herbal preparations of any form, dose, and duration were counted. No intervention, pharmaceutical agents, and placebo were counted in comparison interventions.
(5) Investigations with herbal formulations administered in homeopathic potency or botanicals administered merely in Traditional Chinese Medicine (TCM) were excluded.
(6) Review articles and articles older than 2010 were also not included to include only new and updated studies so that the meta-analysis can be performed.

### 3.2 Outcome Measures

Investigations that evaluated ADHD symptoms such as lack of attention, hyperactivity, and impulsivity, as described by the Diagnostic and Statistical Manual of Mental Disorders or the International Statistical Classification of Diseases and Related Health Problems as a primary or secondary outcome, were taken into consideration as eligibility criteria.

### 3.3 Search Strategy

Electronic databases MEDLINE (PubMed), Scopus, Google Scholar, EMBASE, JSTOR, PsycINFO, CINAHL, and The Cochrane Library were searched from 2010 to 2021 (the systematic review flow chart is depicted in Figure 1). PubMed was used as the primary search engine incorporating search phrases for ADHD combined with particular interventions of herbal remedies or CAMs. Inclusion criteria for the retrieved papers strictly considered open-label studies and human randomized controlled trials (RCTs) depicting sufficient methodological rigor. The search terms were:

(1) ADHD
(2) Medicinal plants OR herbal medicine OR traditional medicine OR plant extracts OR herbal extract OR herbal formulations herb OR Ayurveda OR TCM OR CAM
(3) Clinical trial OR open-label studies OR controlled clinical trial OR randomized clinical trial OR randomized controlled trial OR phase III clinical trial
(4) 1 and 2 and 3

### 3.4 Data Collection

Each retrieved literature was analyzed by scrutinizing the title and abstract. The full texts of only the articles that met the inclusion criteria were adequately assessed. For the investigations that met the selection criteria, the authors independently abstracted the related population and intervention characters with a standardized data extraction format. Any disagreement in this regard was resolved by an exchange of opinions among the authors.

### 3.5 Data Analysis

Meta-analyses were carried out in cases of similar comparison investigations demonstrating similar outcomes. The Odds Ratio (OR) was calculated by the data provided in the publications and their supplementary files using Python 3.3. The Forest plot of the included studies was generated using Python 3.3 and MS PowerPoint based on these ratios. The Funnel plot was constructed by R 4.1.1. The Cochrane analysis was done using the Cochrane risk of bias tool for RCTs in Review Manager 5 software version 5.2.

## 4 Results

### 4.1 Literature Search

**FIGURE 2:**
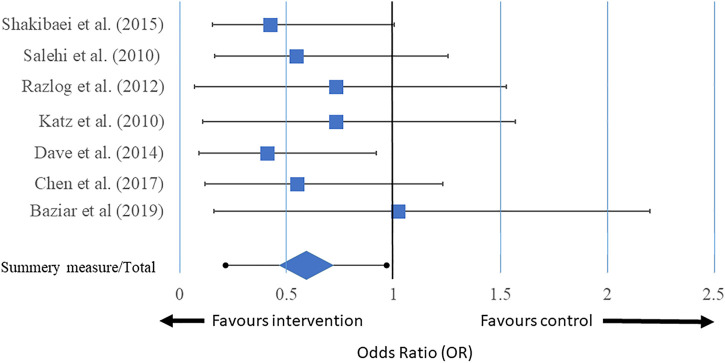
Bar plot of the number of individuals under study in the included works.

**FIGURE 3:**
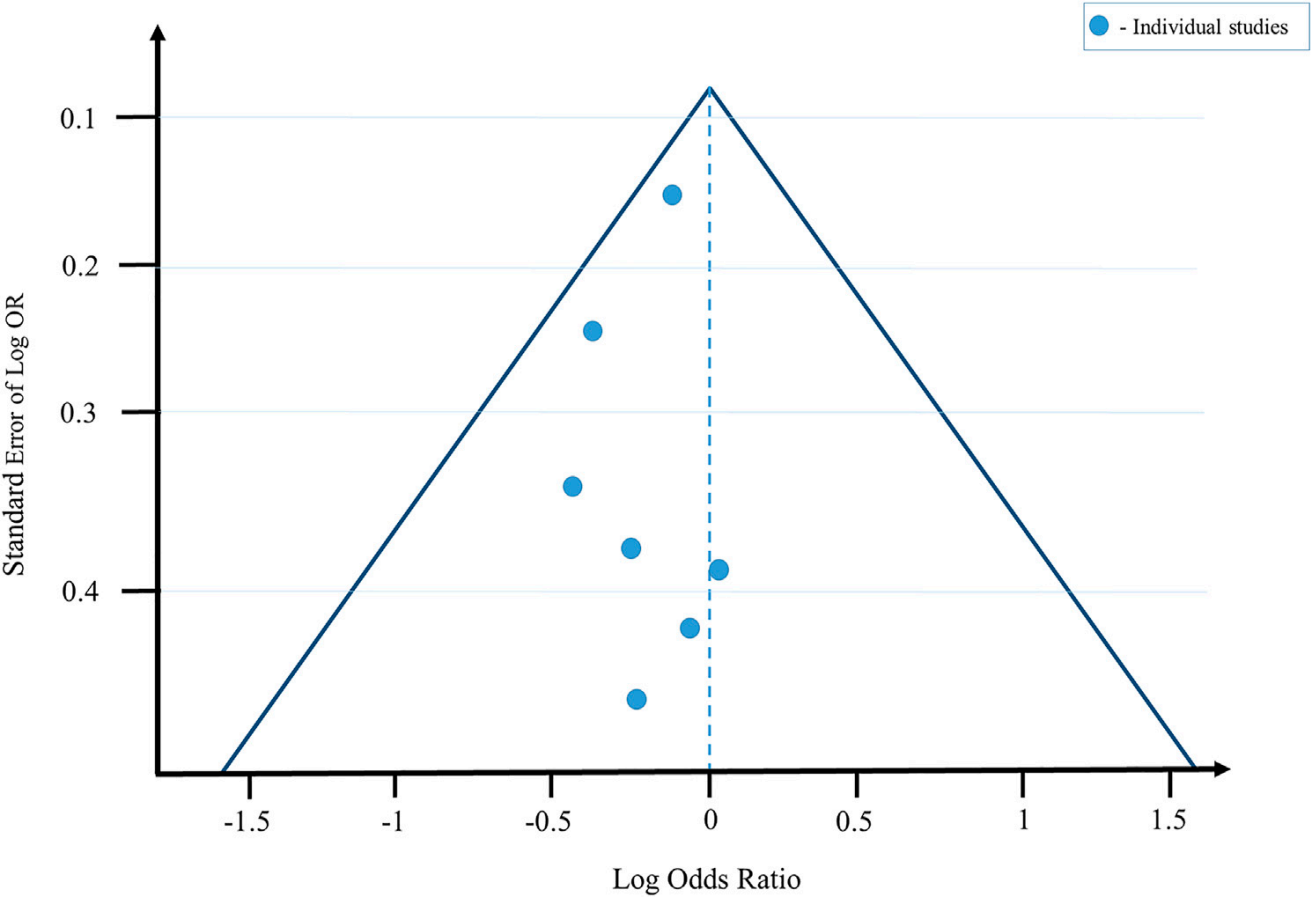
Pie chart of the gender distribution of the participants in the included works.

A total of 1990 non-redundant literature records were obtained through the literature search, out of which 796 were selected after the removal of duplicate studies. 601 articles were rejected following a random screening of the title and abstract where full texts in English were unavailable. 195 full texts were examined for eligibility, from which further 127 articles were excluded since these were not RCTs and did not involve children and adolescents under 18 years of age or were review articles. 68 studies were considered for general review. After excluding 61 publications due to various reasons like study date, symptoms not pertaining to ADHD, 7 studies were included in the evaluation. The PRISMA flow chart for the systemic review is shown in Figure 1. Detailed information on the various parameters of the included studies is presented in Table 1. Table 2 has also been made showing the various active components present in the four plants discussed in the review. The number of individuals under study in individual literature is shown in Figure 2. The gender distribution of participants has been given in Figure 3.

**TABLE 1:** Detailed information of the included studies.

References	Study Design	Study Population	Intervention	Comparator	Measurements	Scores		Outcome
						Parents	Teachers	
Baziar *et al*	Randomised, double-blind, placebo-controlled clinical trial	N = 54	Saffron (*Crocus sativus*) capsules at a dosage of 20–30 mg/d depending on weight (20 mg/d for <30 kg and 30 mg/d for >30 kg)	Methylphenidate at a dose of 0.3–1 mg/kg/day	Symptoms were rated using the Parent and Teacher ADHD-RSIV at baseline and weeks 3 and 6	No significant difference between the two groups on Parent Rating Scale scores.	Changes in Teacher ADHD Rating Scale scores from baseline to the study end were not significantly different between the saffron group and the MPH group	The frequency of adverse effects was similar between saffron and MPH groups
								Short-term therapy with saffron capsule showed the same efficacy compared with methylphenidate
Chen *et al*	Randomised, double-blind, placebo-controlled, cross-over clinical trial	N = 8; 7–16 years of age; gender 7/1 (m/f)	The capsule of extract from pine bark contains 25 mg Oligopin per capsule. 2 interventional periods and one wash-out period of 2 weeks in between	Placebo, which contains 25 mg cellulose	Neuropsychological assessment through Conners’ Continuous Performance Test (CPT-II), measurement of routine blood biochemical parameters and anti-oxidative status	The scores of CPT II by parents showed that the children treated with the pine extract fared better than the placebo group	Decrease in ADHD seen in the students in the treatment group compared to the placebo according to the CPT II assessment by teachers	Administration of the polyphenolic extract for 1 month might improve the inattention and impulsivity and reduce plasma lipid peroxidation levels in children and adolescents with ADHD
Dave *et al*	Open label study	N = 31; 6–12 years of age.	Standardized *Bacopa monnieri* extract (SBME) BacoMind (M/s Natural Remedies, Bangalore, India), 225 mg/d for a period of 6 months		Parent Rating Scale to assess the ADHD symptom scores at baseline, and the team administered it again at the end of the 6 months	SBME significantly reduced the subtest scores of ADHD symptoms, except for social problems. The symptom scores for restlessness were reduced in 93% of children, whereas improvement in self-control was observed in 89% of the children. The attention-deficit symptoms were reduced in 85% of children. Symptom scores for learning problems, impulsivity, and psychiatric problems were reduced for 78, 67, and 52% of children, respectively	Teachers were not included in this study	Treatment with SBME resulted in significant reductions in all subtests of ADHD indicators at 6 months, with the exception of social issues, which saw a nonsignificant reduction in scores
Katz *et al*	Randomised, double-blind, placebo-controlled clinical trial	N = 120; age 6–12 years; gender: exp.grp.: 60/20 contr.grp.: 32/8 (m/f)	3 ml of a compound herbal preparation 3 times a day in 50–60 ml of water (*Melissa officinalis, Paeoniae alba, Withania somnifera, Centella asiatica, Spirulina platensis and Bacopa monieri*)	3 ml placebo 3 times a day in 50–60 ml of water.	TOVA at baseline and post-Treatment. Parent rated daily questionnaire	Significant statistical difference of TOVA scores. Increase of TOVA scores within the experimental groups. No changes in TOVA scores in the control group over the treatment period	Teachers not included	THE CHP did not seem to alleviate symptoms of ADHD
Razlog *et al*	Randomised, double-blind, Placebo-controlled clinical trial	N = 30; age. (5–11 y) gender: 18/9 (m/f).	exp.grp. 1.: *Valeriana officinalis* (mother tincture) 10 drops/3times per day experimental groups 2: *Valeriana officinalis* (homeopathic 3×potency) 10 drops/3 times per day	Placebo tincture in the same dosage	Efficacy was assessed by the Barkley and DuPaul teacher rating scale, the children’s checking task and the parent symptom questionnaire scores at baseline, first and the second week of the treatment period and 1 week after the treatment period	Significant improvements of both treatment groups in nearly all subscales of PSQ but after 2 weeks of treatment	The scores of teacher’s assessment revealed that the treatment group showed a decrease in symptoms of ADHD	Valeriana officinalis MT and 3X may have benefits in the treatment of ADHD, according to findings
Salehi *et al*	Randomised, double-blind, Placebo-controlled clinical trial	N = 50; age: 6–14 years; gender: exp.grp.: 19/6 contr.grp.: 20/5 (m/f)	*Gingko biloba* capsule 80–120 mg/day depending on weight	Methylphenidate 20–30 mg/day depending on weight	External assessment (parents and teacher) at baseline, at day 21 and day 42 of the treatment period	According to parental test scores, the intervention was able to improve the scores in the children	A significant difference between the control and experimental groups show the intervention was effective	Hence *G. biloba* was found to have alleviated symptoms of ADHD
Shakibaei *et al*	Randomised, double-blind, placebo-controlled clinical trial	N = 66; age: 6–12 years; gender: exp.grp.: 19/12 contr.grp.: 20/9 (m/f	*Gingko biloba* enteric coated tablets 80–120 mg per day Depending on weight + usual care (Methylphenidate 20–30 mg per day depending on weight)	Placebo tablets in same dosage + usual care (Methylphenidate 20–30 mg per day depending on bodyweight).	External assessment (parents and teacher) at baseline, week 2 and week 6 of the treatment period; measurement of general psychosocial functioning by a child and adolescent psychiatrist at baseline, week 2 and week 6 of the treatment period	According to parental test scores, the intervention was able to improve the scores in the treated children compared to the placebo	According to teacher test scores, the intervention was able to improve the scores in the treated children compared to the placebo	G. biloba is an effective and safe complementary therapy in the treatment of childhood ADHD. Although the additional effect of the herb on ADHD symptoms was minimal and limited to the inattention symptoms, it resulted in a significant increase in overall clinical treatment response

**TABLE 2:** Active constituents present in the listed plants.

Name of the plant	Active Constituent	Chemical Structure
1. *Bacopa monnieri*	Bacopaside I	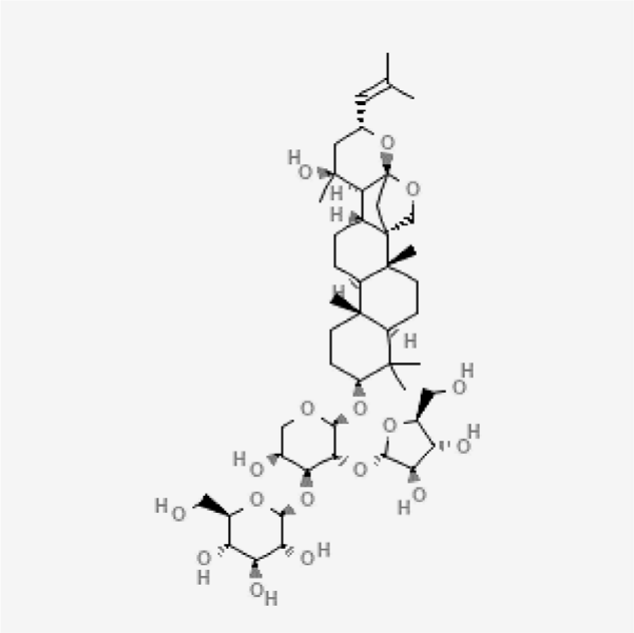
		Bacopaside I
2. *Valeriana officinalis*	a. Valerenic acid	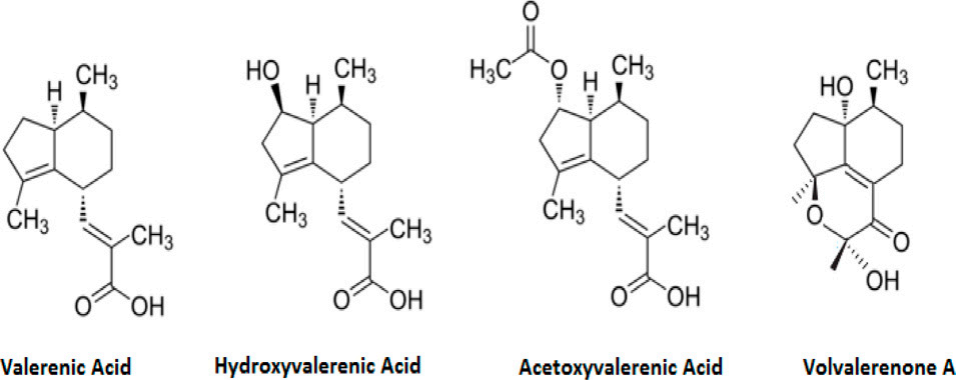
	b. Hydroxyvalerenic Acid	
	c. Acetoxyvalerenic Acid	
	d. Volvalerenone A	
3. *Melissa officinalis*	1. Citronellal	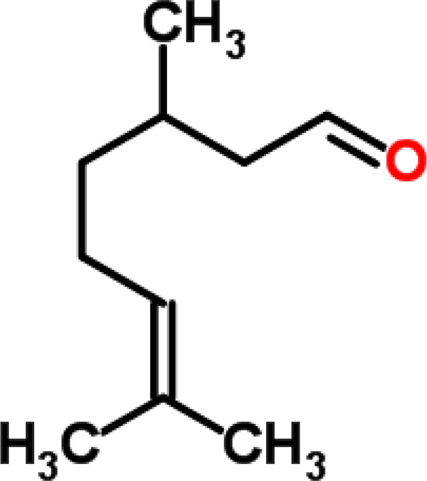
	2. Salvianolic acid	Citronellal
		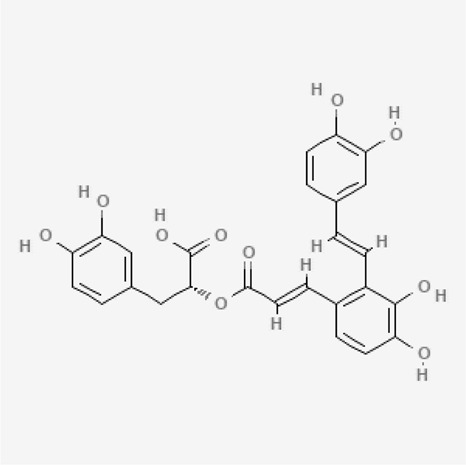
		Salvianolic acid
4. *Ginkgo biloba*	Ginkgolide B	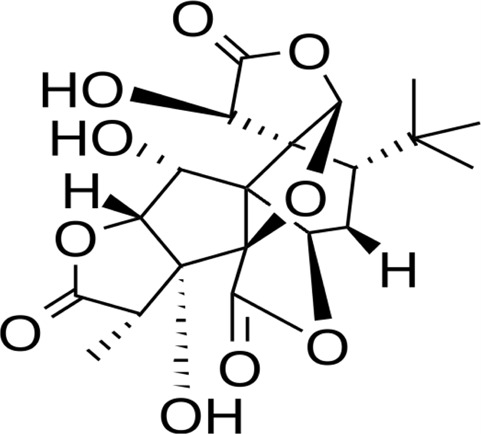
		Ginkgolide B
5. *Pinus pinaster*	Pycnogenol	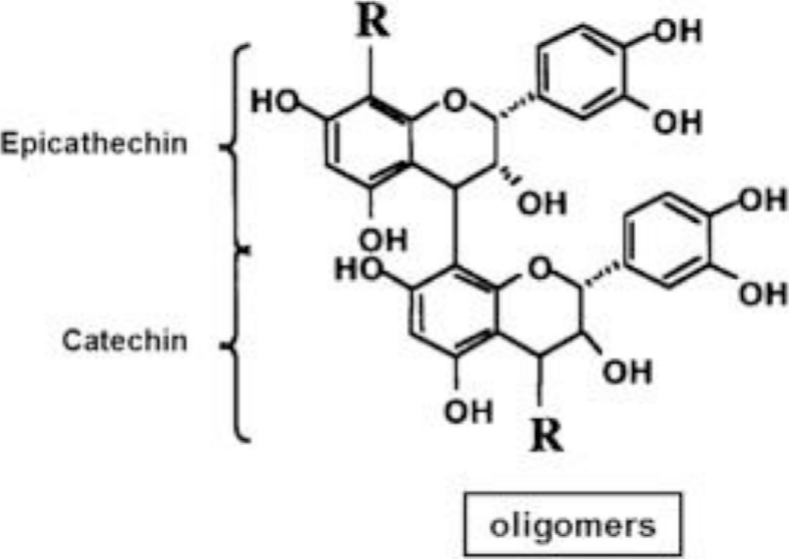
6. *Crocus sativus*	a. Picrocrocin	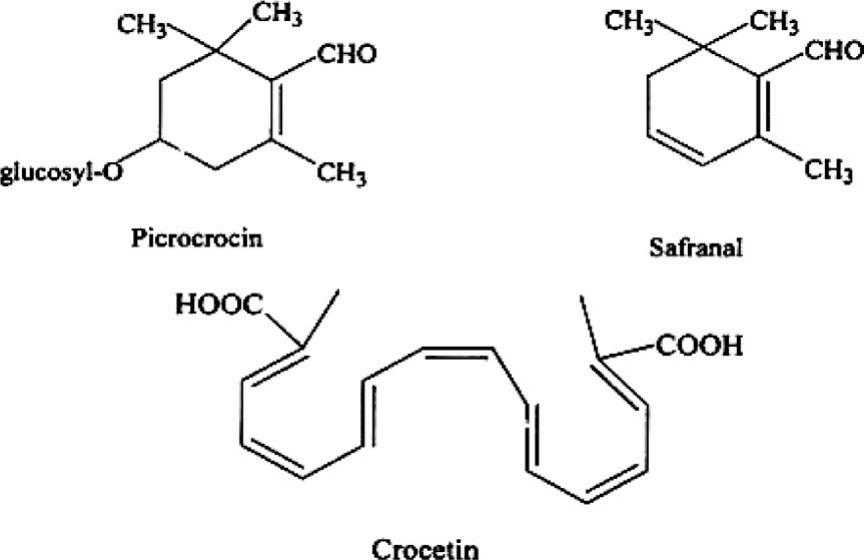
	b. Safranal	
	c. Crocetin	

### 4.2 Meta-Analysis

**FIGURE 4:**
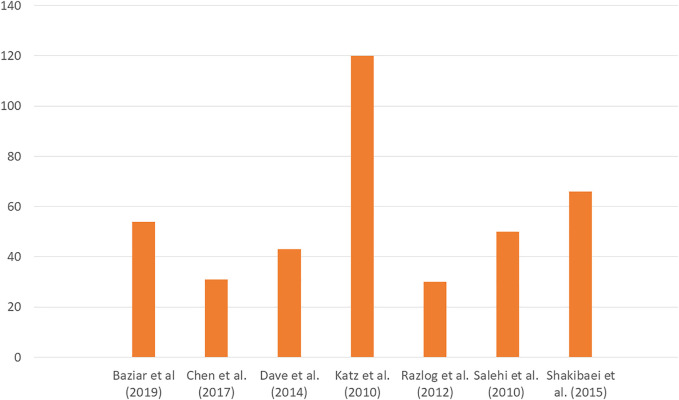
Forest plot of the meta-analysis of individual studies. Generated by Python 3.3.

**FIGURE 5:**
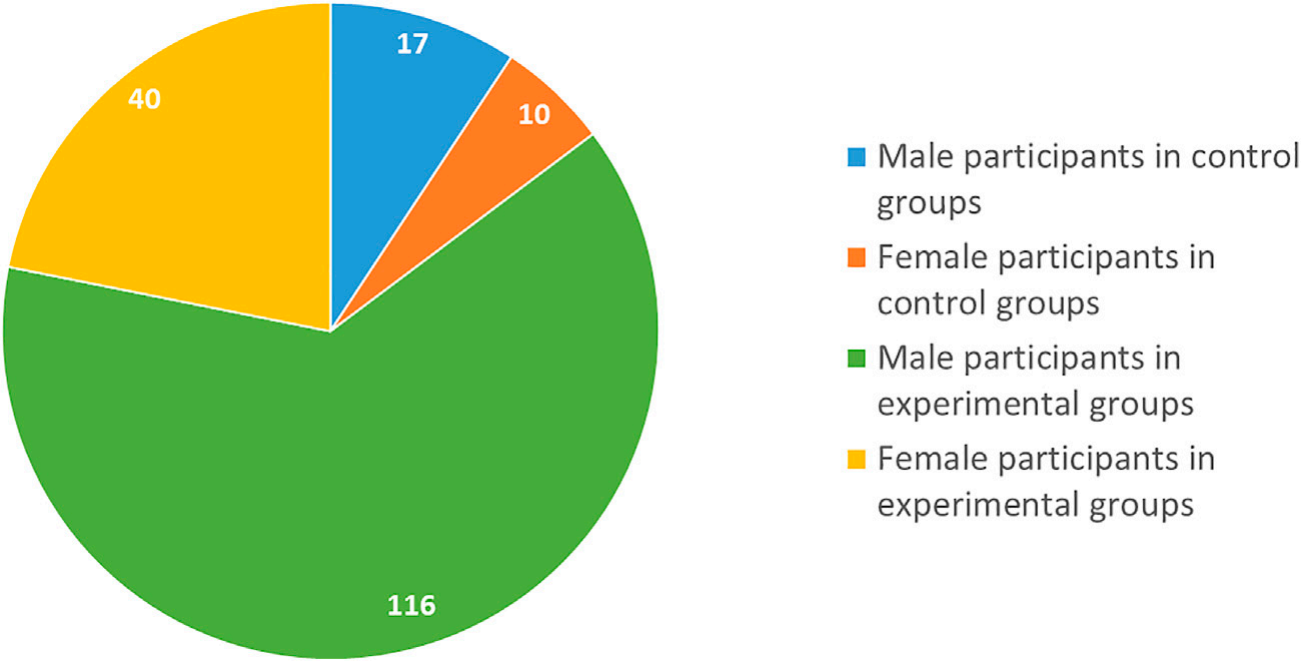
The funnel plot of the meta-analysis shows the biasedness and overall results. Generated by R 4.1.1.

**FIGURE 6:**
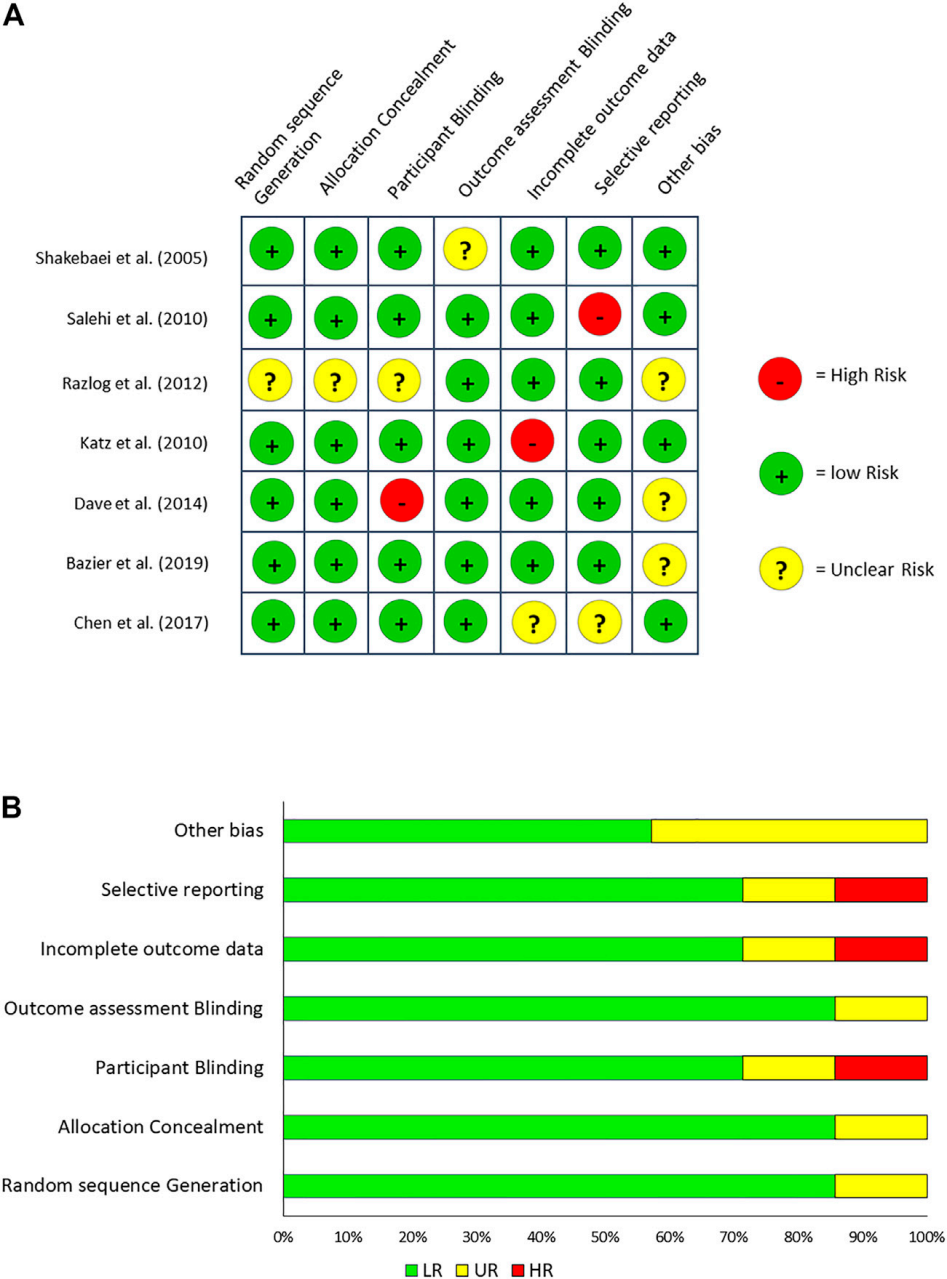
**(A)**: Assessment of risk of bias. **(B)** Risk of bias graph. Key: HR–High Risk, LR–Low Risk and UR–Unclear Risk. Generated with the help of Cochrane risk of bias tool for RCTs in Review Manager 5 software version 5.2.

The forest plot of the individual studies is depicted in Figure 4. The forest plot clearly indicates that only 1 out of 7 were favouring the control. Two of the studies showed no significant differences between the control and treated cohorts. 7 studies favoured treatment with herbal formulations. The overall result of the meta-analysis favoured herbal treatment. The funnel plot is shown in Figure 5 and it showed that the study is asymmetrical and biased towards the favour of treatment. Cochrane analysis of the risk of bias is also done and shown in Figure 6.

### 4.3 Description of Plants, Features of the Included Study and Results

#### 4.3.1 *Ginkgo biloba* L. (Ginkgoaceae)

G. biloba is commonly found in China and is a tall tree highly resistant to harsh weather and has been used as a medicine for ages.

##### 4.3.1.1 Clinical Effects on Various Ailments and Symptoms Different From Attention Deficit Hyperactivity Disorder

*Ginkgo biloba* has been reported to prevent ischemia (Zhang et al., 2012) thus preventing renal failure (Şener et al., 2005), epilepsy (Mazumder et al., 2017) and peripheral nerve damage (Hsu et al., 2004) due to its antioxidant and vasoactive properties (Dey et al., 2014).

##### 4.3.1.2 Neuropsychological Effects

The plant was also found to provide cognitive benefits in patients with Alzheimer’s disease with cholinesterase inhibitor treatment (Dey et al., 2014). Some executive functions, selective attention, and long-term memory were all enhanced by its extracts (Kaschel 2009). It relieves the symptoms of a variety of cognitive illnesses, including vascular dementia (DeKosky et al., 2008) and age-related amnesia (Snitz et al., 2009) as well as psychological ailments ADHD (Sharma et al., 2021), depression (Dai et al., 2018) and anxiety (Singh et al., 2017).

##### 4.3.1.3 Mechanisms of Action

The extracts of G. biloba contain Ginkgolide B, flavonoid-O-glycosides and terpene trilactone which controls H2O2/FeSO4-induced oxidative damage (Shi et al., 2010). It also reduces intracellular ROS generation by preventing amyloid peptide (A)-induced mitochondrial dysfunction and thereby accumulation of the Aβ fibrils which is a potent neurotoxin (Li et al., 2021). Ion homeostasis, modification of tau protein phosphorylation, and activation of growth factor production are all possible pathways for G. biloba extract’s neuroprotective effects (Singh et al., 2019).

##### 4.3.1.4 Features of the Included Study

Here, two studies involving *G. biloba* are included. Salehi et al. (2010) compared a *G. biloba* herbal preparation with methylphenidate against ADHD, whereas Shakibaei et al. (2015) demonstrated an experiment to compare *G. biloba* and placebo as an additional treatment strategy with methylphenidate administration. Both of these studies had an overrepresentation of male participants. The average ages of the trial teams ranged from 9.12 to 38 and 7.83 years, respectively. Documented medication according to the participant’s mass was 80–120 mg for *G. biloba* and 20–30 mg daily for methylphenidate in both investigations. The ADHD rating system IV was used in these analyses to detect ADHD manifestations in caregivers and educators. Salehi et al. (2010) recorded data at the start of the study and on the 21st and 42nd days following the treatment. On the other hand, Shakibaei et al. (2015) took measurements at the start of the study and 2 and 6 weeks after a 6-weeks medication phase. Biasedness was avoided in both tests. Salehi et al. (2010) took serum and urine samples during the sampling interval but findings were not disclosed.

##### 4.3.1.5 Outcomes

In the investigation by Salehi et al. (2010), there were no significant differences in the measured ADHD indicators for the *G. biloba* cohort during the study period. This result was constant for both parental and teacher judgements. Changes in the methylphenidate cohort proved to be considerable for both evaluation circumstances. Additionally, a contrast between the two groups revealed substantial variations in the remission of the disorder. Significant improvements were observed in the children who received methylphenidate but not in the children given *G. biloba*. When the children were given *G. biloba* along with methylphenidate, the conditions improved immensely (Shakibaei et al., 2015). The risk findings of both investigations confirmed only mild to modest complications. In the investigation by Salehi et al. (2010), the disparity in total recurrence of health risks between the groups was not substantial, but sleeplessness, headache, and reduced appetite were identified more frequently in the drug-treated group. Shakibaei et al. (2015) found no significant differences in headache recurrence and other complications between *G. biloba* and placebo. After 14 days of medication, it was discovered that the percentage of participants who improved shown by a drop in T-score for 5 or more points spanned from 31% (anxious-shy characteristic) to 67% (psychosomatic characteristic). The number of cases who improved after a month of treatment ranged from 44% (attribution of social difficulties) to 74% (Conners’ ADHD scale). Only two of the 36 individuals (14%) had significant side effects and only one of them was related to the trial medicine. It was concluded that AD-FX medication may alleviate symptoms of ADHD and that more studies are needed on the utilization of components of ginseng and *G. biloba*.

#### 4.3.2 *Crocus sativus* L. (Iridaceae)

The world’s costliest condiment, saffron or *Crocus sativus*, was traditionally used for its allergy-relieving, antibacterial, analgesic, antitumor, and anticonvulsant properties (Srivastava et al., 2010; Anand et al., 2022). This plant is also used as/against the weakness of local muscular and nervous tissues (Paul et al., 2021). The plant is a “potential” antidepressant and therefore is beneficial for ADHD therapy.

##### 4.3.2.1 Clinical Effects on Various Ailments and Symptoms Different From Attention Deficit Hyperactivity Disorder

Saffron has immunomodulatory (Kianbakht and Ghazavi 2011) and cardioprotective effects (Pourmasoumi et al., 2019). In rats, an aqueous extract of flowers produces a hypotensive effect and prevents atherosclerosis in rabbits (Moshiri et al., 2015). It also helps mediate menstrual pain, erectile dysfunction and skin allergies (Moshiri et al., 2015).

##### 4.3.2.2 Neuropsychological Effects

Crocin and safranal have been proven in cell culture to suppress the development of soluble oligomers and subsequent fibrillar assemblies thus controlling Alzheimer’s and Parkinson’s diseases. It also helps to control major depressive disorder (Moshiri et al., 2015).

##### 4.3.2.3 Mechanisms of Action

Crocins are mono- and diglycosylated esters of the dicarboxylic acid crocetin, which are carotenoids. They account for 3.5 percent of the flower’s overall mass. Picrocrocin (monoterpene glycoside) and safranal (cyclic monoterpene aldehyde) are two chemicals found among carotenoids’ oxidation products (Maggi et al., 2020). Active ingredients serve as receptor inhibitors of N-methyl d-aspartic acid (NMDA) and gamma-aminobutyric acid (GABA) and are suggested to promote the restriction of dopamine and norepinephrine absorption (Sharifi et al., 2020). These components together give protection against ATP induced cytotoxicity by masking the P2X7 receptors (Di Marco et al., 2019). They also mediate intracellular Calcium and thereby protects against oxidative damage which leads to their neuroprotective action (Abdel-Rahman et al., 2020). It also can influence both the monoaminergic and glutamatergic pathways, making it ideal for the treatment of ADHD, which is caused by a breakdown of these pathways (Sarris, 2007; Curatolo et al., 2009).

##### 4.3.2.4 Features of the Included Study

Fifty-four participants between the ages of six to seventeen who suffered from ADHD were arbitrarily medicated with 20–30 mg/d methylphenidate (MPH) or 20–30 mg/d *C. sativus* according to body mass (20 mg/d for 66 lb and 30 mg/d for more than that) in a 1.5 months double-blind study (Baziar et al., 2019). At the beginning, weeks 3 and 6, the syndrome is characterized using the ADHD Rating scale IV by a teacher and a guardian.

##### 4.3.2.5 Outcomes

The test was performed on 54 patients. The *C. sativus* cohort and the MPH cohort did not differ significantly in alterations in both ADHD Rating Scale scores from baseline to the end of the trial. The occurrence of negative effects was comparable in both groups.

#### 4.3.3 *Bacopa monnieri* (L.) Wettst. (Plantaginaceae)

The herb *B. monnieri*, also known as *Herpestis monniera*, water hyssop, and commonly abbreviated as Brahmi in India, has been employed in Ayurveda, an Indian holistic medical approach, for generations (Gohil and Patel 2010).

##### 4.3.3.1 Clinical Effects on Various Ailments and Symptoms Different From Attention Deficit Hyperactivity Disorder

It has analgesic, anti-inflammatory and antipyretic effects (Russo and Borrelli 2005). It alleviates irritable bowel syndrome by helping in smooth muscle movement in the intestines (Russo and Borrelli 2005), It also has vasodilatory effects and mediates blood pressure and helps in respiration (Dar and Channa 1997).

##### 4.3.3.2 Neuropsychological Effects

Several therapeutic and laboratory researches have demonstrated the usefulness of the plant in improving intellect and concentration (Dey et al., 2014). *B. monnieri* has already been shown to help cognitive performance in adolescents with ADHD (Negi et al., 2000).

##### 4.3.3.3 Mechanisms of Action

The active ingredient, Bacoside I has neuropharmacological characteristics and pseudo-jujubogenin units, also referred to as aglycone monomers (dammarane-type triterpenoid saponin). Bacosides inhibit Aß aggregation and fibril formation and protect neurons from Aß induced damage (Abdul Manap et al., 2019). Bacosides can cross the blood-brain barrier and hence mediate the mechanism of GABA and NMDA receptors (Piyabhan et al., 2019). Depending on the testosterone hydroxylase catalytic activity in the liver and gut, it reduced intestinal Pgp and CYP3A expression (Sukumaran et al., 2019). These reduce brain inflammation and improve memory.

##### 4.3.3.4 Features of the Included Study

The open-label trial included 31 children aged 6–12 years, who showed symptoms before the age of seven according to the criteria of the Diagnostic and Statistical Manual of Mental Disorders (DSM-IV) for ADHD. For 6 months, the children received standardized *B. monnieri* extract (SBME) at a level of 225 mg/d. BacoMind (M/s Natural Remedies, Bangalore, India) provided the SBME used in the investigation.

##### 4.3.3.5 Outcomes

The investigators used the Parent Rating Scale to assess ADHD symptoms at the beginning and the end of the 6-months study duration. Except for social issues, SBME dramatically lowered the subtest ratings of ADHD symptoms (Dave et al., 2014). In 93% of the children, the hyperactivity trait scores were reduced, while consciousness improved in 89% of the individuals. The signs of the disorder were alleviated in 85% of the individuals. Similarly, for 78% 67%, and 52% of the group, trait scores for learning problems, impulsivity, and mental problems, respectively, decreased.

#### 4.3.4 *Pinus pinaster* Aiton (Pinaceae)

Also known as maritime pine, the Mediterranean pine is a robust, medium-sized, rapid growing conifer found commonly on western Mediterranean coasts. Its aqueous extract Pycnogenol is used for medicinal purposes.

##### 4.3.4.1 Clinical Effects on Various Ailments and Symptoms Different From Attention Deficit Hyperactivity Disorder

It helps attenuate chronic obstructive pulmonary disease by mediating the ERK SP1 pathway (Shin et al., 2016). It shows hypoglycaemic and anti-microbial properties in laboratory models (Ferreira-Santos et al., 2020). It also has wound healing and anti-inflammatory properties (Tümen et al., 2018).

##### 4.3.4.2 Neuropsychological Effects

Pycnogenol improves memory and spatial learning in rodents (Afşin et al., 2017) as well as slows down cognitive aging in humans (Simpson et al., 2019). It serves as a neuroprotective via the ERK1/2 MAPK pathway (Xia et al., 2017).

##### 4.3.4.3 Mechanisms of Action

Flavonoids, proanthocyanidins, catechins, and phenolic acids are abundant in the fruit. Pycnogenol, a nutritional additive made from *P. pinaster* bark residues, is advertised as a treatment for a variety of ailments. It contains 70% procyanidin, a powerful antioxidant (Schoonees et al., 2012). Other constituents and enzyme enhancers include catechins, biflavonoids, and phenolic acids (Dvoráková et al., 2006). Pycnogenol inhibits nitric oxide production and inducible nitric oxide synthase (iNOS) upregulation thus decreasing inflammation (Uhlenhut and Högger 2012). It is also capable of crossing the blood–brain barrier (BBB) via the GLUT-1-type glucose transporter and decreases beta-amyloid activity (Simpson et al., 2019). It also mediates lipid metabolism via TLR4–NF-κB pathway (Luo et al., 2015).

##### 4.3.4.5 Features of the Included Study and Outcomes

Chen et al. in a randomized placebo (25 g cellulose) controlled test on 8 children between 7 and 16 years of age found that administration of capsules containing *Pinus* extracts improved lack of attention and restlessness. The results were concluded with the help of Conner’s Continuous performance test (CPT II).

Two experimental periods were followed by a 2-week washout interval. Conners’ Continuous Performance Test (CPT-II) was used to assess neurocognitive function as well as monitor regular blood physiological markers and the level of antioxidants. In children and adolescents with ADHD, taking a polyphenolic isolate for 30 days may help with lack of attention and hyperactivity, while also lowering plasma lipid peroxidation concentrations.

#### 4.3.5 *Melissa officinalis* L. (Lamiaceae)

Lemon balm or Melissa *officinalis* is a shrub native to southern Europe named after its lemon-like fragrance (Chan et al., 2000). It is used both as a flavouring agent in cooking as well as a medicinal herb.

##### 4.3.5.1 Clinical Effects on Various Ailments and Symptoms Different From Attention Deficit Hyperactivity Disorder

It is used against gastrointestinal disorders as a sedative and antispasmodic drug (Ozarowski et al., 2016). It serves as a nutritional supplement for cardioprotection (Draginic et al., 2021) as well as a hepatoprotective agent which mediates cholesterol (Bolkent et al., 2005). Leaf extracts are used in the treatment of herpes (Miraj et al., 2016).

##### 4.3.5.2 Neuropsychological Effects

It serves as an anti-anxiety (Cases et al., 2011) and helps relief ADHD in both children (Anheyer et al., 2017) and adults (Chan et al., 2000). It has been used in phytotherapy for the prevention and treatment of nervous system problems such as insomnia (Ozarowski et al., 2016).

##### 4.3.5.3 Mechanisms of Action

The active component is citronellal and salvianolic acid which is an anti-oxidant and anti-microbial (Stojanović et al., 2022). It inhibits the MAPK pathway and hence shows a neuroprotective function. Amyloid beta-protein aggregation, fibril formation, 1-methyl-4-phenylpyridin-induced neurotoxicity and amyloid beta-cellular protein toxicity is reduced by the components (Ozarowski et al., 2016).

##### 4.3.5.4 Features of the Included Study

Katz et al. (2010) investigated a complex botanical preparation as an alternate therapy for ADHD indicators in 120 individuals. *Melissa officinalis* was the principal component in this mixture, but it also included Ayurvedic and other herbal extracts such as *Bacopa monnieri*. The therapeutic dose was fixed at 3 ml per 50–60 ml of water. The children ranged in age from 6 to 12 years old. This research included an overabundance of male subjects without making mention of racial distinctions. The key outcome indicator was the result of TOVA or the test of attention variables. There was a substantial chance of missing study results for more than half of the exclusions of the placebo cohort.

##### 4.3.5.5 Outcomes

The TOVA score of the experimental group improved significantly between the initial and final measurements. The TOVA’s evaluations and aggregated results demonstrated significant modifications. However, there were no substantial changes in the placebo cohort during the test period. There were also huge discrepancies between the CHP (compound herbal preparation) and placebo groups. Apart from mild secondary effects such as headaches, there was no indication of any major health risks. Well-accepted CHP was shown in the treatment cohort to increase focus, intelligence, and pulse regulation, suggesting possibilities in children for therapy with ADHD.

#### 4.3.6 *Valeriana officinalis* L. (Caprifoliaceae)

Valerian or *Valeriana officinalis* is a perennial species which bears fragrant pinkish flowers. It is found in Asia, North America and Europe and is used for easing sleep because of its pleasant fragrance (Leathwood et al., 1982).

##### 4.3.6.1 Clinical Effects on Various Ailments and Symptoms Different From Attention Deficit Hyperactivity Disorder

It is used as an antispasmodic, diuretic, anthelmintic, diaphoretic, and emmenagogue (Murti et al., 2011). It also helps in improving digestion and prevents Irritable bowel movement (Nandhini et al., 2018). It inhibits acute coronary insufficiency and arrhythmia caused by vasopressin, as well as having modest positive inotropic and negative chronotropic effects (Chen et al., 2015).

##### 4.3.6.2 Neuropsychological Effects

It has soothing and mood-lifting qualities that have a profound benefit on the CNS (Central nervous system). In recent times, ADHD and comparable conditions characterised by restlessness have been shown to be relieved by this species, which is commonly used to manage diseases including stress and sleeplessness (Balch 2002).

##### 4.3.6.3 Mechanisms of Action

Active components include Valerenic acid, Hydroxyvalerenic Acid, Acetoxyvalerenic Acid and Volvalerenone A (Patočka and Jakl 2010). These components react with GABA receptors to promote cortical excitability. By forming a complex with SUR1, it regulates potassium ions in the pancreas by reacting with KIR 6.2 a potassium sensitive channel. This modifies the action of the enzyme tyroxine hydrolase thereby increasing dopamine (Santos et al., 2016).

##### 4.3.6.4 Features of the Included Study

Razlog et al. (2012) conducted a pilot RCT to see if *V. officinalis* had any effect on the symptoms of ADHD. Two results were matched, one receiving an infusion of Valeriana officinalis and the other receiving a homoeopathic strength of this medication. A placebo decoction was used for the controlled group. Only the evaluation of the original infusion and placebo was considered relevant for the purpose of the review. The 30 participants ranged in age from 5 to 11, with males slightly outnumbering females. ADHD indicators were measured at the beginning of the study, followed by subsequent measurements for the next 2 weeks. The final measurement was taken 2 weeks after treatment. During the PSQ (Conner’s parent symptoms questionnaire), the CCT (children’s check task) and the Barkley and DuPaul teacher rating scale, personal and objective evaluations were discovered. No more observations were made. The existence of choice, productivity, recognition, and other bias created uncertainty.

##### 4.3.6.5 Outcomes

With the exception of psychosomatic difficulties and behavior disorders, the PSQ demonstrated an increase in virtually all subscales compared to the control in both intervention groups. Advancements were observed after 2 weeks of medication, but not after the first week of follow-up. After the first and second weeks after the intervention, both test groups showed a significant improvement in the overall score and the pace score of the CTT evaluation. There was no considerable improvement in the control group. After 2 weeks of therapy, both cohorts receiving *V. officinalis* showed significant progress on the 9/14 subscales of the Barkley and DuPaul teacher evaluation scale. Only 2/14 questions were performed significantly better in the control group. The difference between the test and control groups was statistically significant. The authors reported no major adverse events or health consequences.

## 5 Discussion

### 5.1 Brief Summary

Only a few herbal formulations were found to have little benefit evidence in a systematic analysis of 7 RCTs on herbal therapeutic approaches in 200 children with ADHD. The meta-analysis shows that the studies significantly favor the treatment with herbal medicines. Cochrane analysis shows there is overall a low risk of bias. Several therapy options involving *M. officinalis* *, B. monnieri,* and *V. officinalis* improved many metrics in self-administered cognitive and psychometric abilities; however, the results in externally administered questionnaires were debatable. As a compound herbal remedy, *M. officinalis* and *G. biloba* were combined with *Panax quinquefolius*, but the results must be read more critically. Furthermore, Trebatická et al. (2006) found that minors administered pine bark isolate improved in particular teacher-rated subtests but not in parental-rated surveys. The effectiveness of *C. sativus* in ADHD therapy was nearly non-existent, while the infusion findings of *G. biloba* and pine bark were ambiguous. No potential adverse effects were discovered.

### 5.2 Concurrence With Previous Studies

The results of this study are consistent with those of a previous review of the literature that looked at the proof for herbal treatments in the management of ADHD. For the various herbal remedies, similar results were achieved (Pellow et al., 2011; Lopresti 2015; Anheyer et al., 2017).

### 5.3 Limitations

For any scientific work, there are various associated limitations. Similarly, here there are also different limitations to taking a note. Although the screening technique was thorough at first, there is still a chance that relevant unpublished articles or publications in languages other than English were overlooked inadvertently. As a result, linguistic and publication bias cannot be eliminated. Furthermore, the absence of data on inclusion criteria is a crucial constraint of this assessment. Except for a few herbs, such as *P. pinaster* and *G. biloba*, most medicinal herbs have single publications.

Furthermore, the limited sample sizes of the research must be deemed a drawback in *G. biloba*. Also, in most studies, there is an overrepresentation of male candidates, which does not portray a clear idea for both genders. Therefore, it is still too early to definitively conclude the efficacy and safety of the different herbal treatments at this time. Further investigation and detailed studies can provide conclusive evidence in this field.

Sonuga-Barke et al. (2013) in order to deal with the issue of the potential for bias due to lack of blinding is psychosocial interventions tarted to compared the so-called the “most proximal” outcome (MPROX—i.e., rated by persons closest to treatment delivery and, therefore, the most vulnerable to lack of blinding) with the measure judged by the group consensus to be most blinded (PBLIND, i.e., probably blinded). In a series of meta-analyses, the same working group (European Network for Hyperkinetic Disorders-EUNETHYDIS; Daley et al., 2014; Stevenson et al., 2014; Cortese et al., 2016; Coghill et al., 2021) showed that in non-pharmacological trials, MPROX effects were considerably larger (and more significant) than PBLIND effects though the scale of this MPROX-PBLIND discrepancy varied by treatment type—largest for parent training (where blinding was most challenging to implement) and smaller (though still substantial) for neurofeedback and cognitive training. An overview of systematic reviews of dietary interventions concluded that individual study methods were weak and that different meta-analyses have used very different inclusion and exclusion criteria and that this has resulted in a wide range of estimated effect sizes (Stevenson et al., 2014; Banaschweski et al., 2018). There was a small but statistically significant effect on probably blinded ratings for supplementation with free fatty acids, while the evidence to support either restricted elimination diets or elimination of artificial food colors was significantly less certain.

## 6 Conclusion

There is a fair and satisfactory indication of the effectiveness of *M. officinalis* as an element of a CHP. *Bacopa monnieri, Matricaria chamomilla,* and *Valeriana officinalis* from the studies evaluated in this systematic review to manage specific manifestations of ADHD. Both herbal preparations provided satisfactory results. Trials with these herbal therapies also point to the relative safety of the medicines with no severe side effects except for one side effect. In general, there are not enough RCTs looking into herbal therapies for ADHD, which does not allow for making any solid recommendations for use at this point.

Nevertheless, obviously at this preliminary and exploratory point, the positive benefit-risk ratio involved *M. officinalis* as a constituent of a CHP, *Bacopa monnieri*, and *Valeriana officinalis* call for their recommendation for use as an alternative treatment. The funnel plot is asymmetrical and biased towards herbal treatment, and 6 out of 7 individual studies in the forest showed that treatment with plant-based medicines significantly decreased symptoms associated with ADHD. Hence, the meta-analysis concluded that herbal formulations could be an effective treatment for the control of ADHD.

Currently, there is not enough data to establish that herbal remedies are very effective as complementary and alternative medicines (CAM) for minors affected by ADHD. Some of the featured trials had weak or ambiguous qualitative characteristics (with unknown risk of bias); therefore, more stringent, higher-quality RCTs are needed in this field. Additionally, it is necessary to learn more about the long-term tolerability and effectiveness of medicinal herbs for ADHD and the effects of various dosage, harvest, and manufacturing factors on health outcomes. As anti-ADHD treatment is mainly based on human samples, especially children, rigorous preclinical and clinical trials are needed in the anti-ADHD drug discovery program.

## Data Availability

The original contributions presented in the study are included in the article/Supplementary Material, further inquiries can be directed to the corresponding authors.
