# Corrigendum: Phytotherapy for attention deficit hyperactivity disorder (ADHD): a systematic review and meta-analysis

**DOI:** 10.3389/fphar.2024.1365806

**Published:** 2024-03-14

**Authors:** Tusheema Dutta, Uttpal Anand, Shreya Sikdar Mitra, Mimosa Ghorai, Niraj Kumar Jha, Nusratbanu K. Shaikh, Mahipal S Shekhawat, Devendra Kumar Pandey, Jarosław Proćków, Abhijit Dey

**Affiliations:** ^1^ Department of Life Sciences, Presidency University, Kolkata, India; ^2^ CytoGene Research & Development LLP, Lucknow, Uttar Pradesh, India; ^3^ Department of Biotechnology, School of Engineering & Technology, Sharda University, Greater Noida, India; ^4^ Department of Pharmaceutical Chemistry, Smt N. M. Padalia Pharmacy College, Ahmedabad, India; ^5^ Department of Plant Biology and Biotechnology, Kanchi Mamunivar Government Institute for Postgraduate Studies and Research, Lawspet, India; ^6^ Department of Biotechnology, School of Biosciences, Lovely Professional University, Phagwara, India; ^7^ Department of Plant Biology, Institute of Environmental Biology, Wrocław University of Environmental and Life Sciences, Wrocław, Poland

**Keywords:** herbal treatments, attention deficit hyperactivity disorder (ADHD), complementary alternative medicine (CAM), phytotherapy, *Melissa officinalis* L., *Valeriana officinalis* L

In the published article, there was an error in the Affiliation information about Uttpal Anand (affiliation 2). Instead of “Department of Life Sciences, Ben-Gurion University of the Negev, Beer-Sheva, Israel,” it should be “CytoGene Research & Development LLP, Lucknow, Uttar Pradesh, India.”

As a result of the change in affiliation above, the Conflict of Interest statement must be amended to the following.

